# Use of Near-Infrared Spectroscopy to Discriminate DFD Beef and Predict Meat Quality Traits in Autochthonous Breeds

**DOI:** 10.3390/foods11203274

**Published:** 2022-10-20

**Authors:** David Tejerina, Mamen Oliván, Susana García-Torres, Daniel Franco, Verónica Sierra

**Affiliations:** 1 Centro de Investigaciones Científicas y Tecnológicas de Extremadura (CICYTEX-La Orden), Junta de Extremadura, Guadajira, 06187 Badajoz, Spain; 2 Servicio Regional de Investigación y Desarrollo Agroalimentario (SERIDA), Carretera AS-267, PK 19, 33300 Villaviciosa, Spain; 3 Instituto de Investigación Sanitaria del Principado de Asturias (ISPA), Avda. Roma s/n, 33011 Oviedo, Spain; 4 Centro Tecnológico de la Carne de Galicia, Rúa Galicia N° 4, Parque Tecnológico de Galicia, 32900 San Cibrao das Viñas, Spain; 5 Department of Chemical Engineering, Campus Vida, Universidade de Santiago de Compostela, 15782 Santiago de Compostela, Spain

**Keywords:** NIRS, Asturiana de los Valles, Rubia Gallega, Retinta, meat quality, DFD classification

## Abstract

The potential of near-infrared reflectance spectroscopy (NIRS) to discriminate Normal and DFD (dark, firm, and dry) beef and predict quality traits in 129 Longissimus thoracis (LT) samples from three Spanish purebreeds, Asturiana de los Valles (AV; *n* = 50), Rubia Gallega (RG; *n* = 37), and Retinta (RE; *n* = 42) was assessed. The results obtained by partial least squares-discriminant analysis (PLS-DA) indicated successful discrimination between Normal and DFD samples of meat from AV and RG (with sensitivity over 93% for both and specificity of 100 and 72%, respectively), while RE and total sample sets showed poorer results. Soft independent modelling of class analogies (SIMCA) showed 100% sensitivity for DFD meat in total, AV, RG, and RE sample sets and over 90% specificity for AV, RG, and RE, while it was very low for the total sample set (19.8%). NIRS quantitative models by partial least squares regression (PLSR) allowed reliable prediction of color parameters (CIE L*, a*, b*, hue, chroma). Results from qualitative and quantitative assays are interesting in terms of early decision making in the meat production chain to avoid economic losses and food waste.

## 1. Introduction

The occurrence of dark, firm, and dry (DFD) meat is a problem in the beef sector. DFD meat looks unappealing and tends to be rejected by consumers, and also has poorer processing characteristics, a lower yield, and higher spoilage potential compared to normal meat, causing important economic losses [1]. This problem is common in many countries, and different occurrence rates have been reported (from 1 to 50% of carcasses) depending on seasonal and geographic variations [2].

Meat producers in Spain have promoted the development of native bovine breeds in the meat production industry, as they are better adapted to the heterogeneous regional production systems and are increasingly in demand by consumers, not only on account of their sensory quality aspects, but also because of the increase in local trade as a sustainability strategy. Nevertheless, in Spain 13.9% of beef still has a pH higher than 5.8 at 24 h postmortem (pH24) [3], which is a major problem in the meat industry.

One of the main factors related to the occurrence of DFD meat is stress associated with multiple factors, including poor nutrition, the animal’s age, duration of transport to the slaughterhouse, environmental temperature, animals fighting and overcrowded lairage, the processing systems of slaughtering plants, the temperament and aggressiveness of animals, and, to some extent, subclinical diseases. Under these conditions, glycogen reserves are depleted before slaughter, which reduces the available glycogen in the muscle, causing an abnormal increase in pH, usually to values over 5.8. This high pH affects meat quality traits, with increased water holding capacity (WHC) and a dark color (lower lightness) that is rejected both by retailers and consumers. In addition, dark cuts are more susceptible to bacterial spoilage, which reduces their beef flavor and causes an anomalous tenderization process.

In beef, pH24 values are considered normal in the range of 5.4 to 5.8 [4], and pH24 ≥ 6.0 has been considered the main indicator of DFD meats [5]. However, depending on the species and the different muscles of the carcass, even higher pH24 values (over 6.2) should be considered [6]. Therefore, pH is not always a sufficiently reliable indicator to clearly identify DFD meat [7], and other aspects related to meat quality should be considered. In fact, several studies have suggested combinations of pH, color, WHC, and Warner–Braztler shear force (WBSF) values to properly classify DFD samples [8]. However, no reliable methodology has been established to allow fast and early identification of DFD meats within the meat chain.

Near-infrared reflectance spectroscopy (NIRS), a sensitive and non-destructive technology that does not require reagents or produce waste, provides comprehensive information about the molecular bonds of organic and chemical constituents and the tissue ultra-structure in a scanned sample. Therefore, is a convenient tool not only for the characterization of foods, but also for the performance of quality measurements and process control procedures [9]. These characteristics, and the fact that it needs minimal or no sample preparation, make NIRS an ideal tool for the quantitative estimation of major constituents in meat and meat products such as fat, protein, moisture, and fatty acids, as well as technical aspects (pH, color, WHC, WBSF) and sensory attributes [10,11]. Moreover, NIR has also been used for classifying animals according to age, diet, breed, or meat freshness [11].

In recent years, the potential of NIRS technology for discriminating defective meats has also been analyzed [8,12,13,14].

Therefore, the purposes of this work were to evaluate the potential of NIRS technology to discriminate between Normal and DFD beef from three purebreeds, Asturiana de los Valles (AV), Rubia Gallega (RG), and Retinta (RE) and to assess its capacity for early prediction (spectra taken 24 h postmortem) of meat quality traits associated with DFD meat at different aging times in order to enable decision making on meat quality.

## 2. Materials and Methods

### 2.1. Animals and Meat Sampling

A total of 129 yearling bull carcasses from 3 autochthonous purebreeds, Asturiana de los Valles (AV; *n* = 50), Rubia Gallega (RG; *n* = 37), and Retinta (RE; *n* = 42), were randomly selected from different slaughtering batches. The animals were stunned with a captive bolt, slaughtered, and dressed according to current EU regulations (Council Directive 93/119/EC; OJ, 1993) in authorized abattoirs. The carcasses were chilled in a conventional chamber at 2 °C (relative humidity 98%). At 24 h postmortem, the Longissimus thoracis et lumborum (LTL) muscle was removed from the left half of the carcass between the 6th and 10th ribs and cut into several 3.5 cm steaks, which were used for pH24, instrumental colour (after 60 min blooming), and drip loss determination and NIR spectra collection.

### 2.2. Meat Quality Measurements

At 24 h postmortem, the temperature and pH of LTL were recorded at the level of the 6th rib using a penetration electrode coupled with a temperature probe (InLab Solids Go-ISM, Mettler-Toledo S.A.E., Barcelona, Spain), which has a spear-shaped tip designed for piercing solid and semi-solid food samples. The electrode was inserted perpendicularly in the carcass (depth of 25 mm) and the pH and temperature were recorded. The average pH value of triplicate measurements was used to categorize the carcasses into 2 quality groups: Normal (pH24 < 6) and DFD (pH24 > 6). Meat color of three 10 mm diameter spots on the exposed cut surface of the steak was recorded at 24 h postmortem using a portable Minolta CM-2300d spectrophotometer (Konica Minolta Inc., Osaka, Japan) under fixed conditions with C and D65 illuminant, 10° standard observer angle geometry, and 8 mm aperture size in the CIE space [15]. The average value of the 3 spots was used. Indicators of lightness (L*), redness (a*), yellowness (b*), chroma (C* = √(a*^2^ + b*^2^)), and hue (h* = tan − 1b*/a*) were measured after 60 min of blooming.

Meat drip loss (% exudate) was determined by duplicates on 50 g fresh samples taken at 24 h postmortem and placed in a special container (meat juice collector, Sarstedt, Germany), for 24 h at 4 °C. Meat drip loss (% exudates) was determined according to Honikel’s method [16]. The drip loss percentage was calculated by the gravimetric difference and expressed as a percentage (g of water released per 100 g of muscle).

### 2.3. NIR Spectra Collection and Spectral Pretreatment

**Figure 1:**
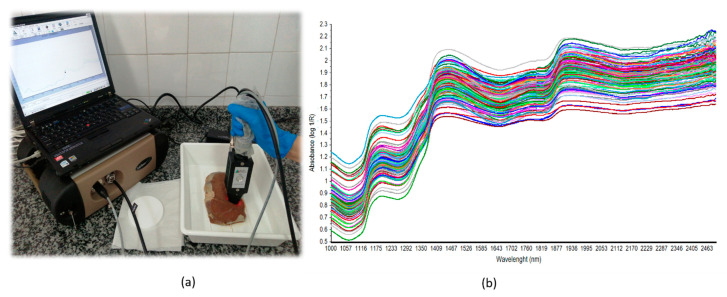
NIR spectrum acquisition system. (**a**) LabSpec 5000 fitted with a fiber optic contact probe (ASD Inc., Boulder, CO, USA); (**b**) absorbance spectra of all samples included in the study (1000–2500 nm). Different color lines represent each different spectra.

Reflectance spectra were collected using a LabSpec 5000 NIR spectrometer with an ASD fiber optic Contact Probe^®^ (21 mm window diameter) (ASD Inc., Boulder, CO, USA). The LabSpec spectrometer optimizes the detector sensitivities for the probe and light source being used, and the integration time was set as 17 ms. The spectrometer takes and subtracts the dark current on every scan, and prior to spectral acquisition, the instrument was calibrated using a Spectralon tile as the white reference. Two spectra per steak (3.5 cm thick) were collected by scanning the intact surface in a zigzag pattern, in order to increase the area of muscle scanned and reduce the sampling error [9]. Each reflectance spectrum was obtained from an average of 40 scans over a range of 1000 to 2500 nm (Figure 1).

Instrument control and initial spectral manipulation were performed using the Indico TM Pro software package (ASD Inc., Boulder, CO, USA). The data were subsequently imported into Unscrambler X v.10.5 (CAMO^®^, Trondheim, Norway) for chemometric analysis.

Outlier spectra were identified during model development and removed. The criteria for deleting outliers were as follows: (1) samples with studentized residual higher than 3, and (2) samples with large leverage, higher than 3 times the average, with the average leverage calculated according to Faber [17] as:

$$H=\frac{1}{n+\frac{number\;of\;principal\;components}{n}}$$

where *n* is the number of samples.

The spectra were first converted from reflectance to absorbance (log 1/R), because this has a more linear response with the concentration of organic compounds. Subsequently, spectra were preprocessed in order to reduce scattering effects and heighten the signals related to the organic compounds in beef samples using various methods. The methods included standard normal variate (SNV), used for correct path length, scattering effects, source or detector variations, and some instrumental sensitivity effects, and removing noise from the spectra; detrend correction (DE); and first- or second-order Savitzky–Golay (SG) derivatives, which can remove both additive and multiplicative effects and reduce insignificant baseline signals from samples. SG is the most popular method, as it includes a smoothing step to reduce the signal-to-noise ratio. Specifically, the first-order derivative with 4 smoothing left- and right-side points and first polynomial order (1,4,4,1) and the second-order derivative with 5 smoothing left- and right-side points and second polynomial order (2,5,5,2) were used. Combinations of these methods (SNV-DE; SG 1,4,4,1-SNV; SG 1,4,4,1-SNV-DE; SG 2,5,5,2-SNV; and SG 2,5,5,2-SNV-DE) were also tested. Moreover, to test the hypothesis that more robust models could be developed for specific traits by selecting specific ranges of the spectrum, two separate subset wavelength ranges were used: the full spectrum (1000–2500 nm) or a bounded spectral range (1000–1800 nm) including only the data collected in the first NIR detection by the LabSpec 5000.

### 2.4. Chemometric Analysis

Descriptive statistics for meat quality traits (pH24, color, and drip loss) were calculated. The outliers were removed, and the variables were tested for normal distribution using Unscrambler X v. 10.5 software (CAMO^®^, Trondheim, Norway).

The average pH value of triplicate measurements was used to categorize the carcasses into 2 quality groups: Normal (pH < 6; *n* = 111) and DFD (pH > 6; *n* = 18) beef samples.

#### 2.4.1. NIR Classification (Normal vs. DFD)

Two qualitative analyses were carried out: partial least squares-discriminant analysis (PLS-DA) and soft independent modelling of class analogies (SIMCA).

##### Partial Least Squares-Discriminant Analysis (PLS-DA)

The PLS-DA algorithm correlates spectral variations and category classes and attempts to maximize the covariance between them. The independent variables (X) are the spectra of each sample, while the dependent variable (Y) is a categorical variable defined by the analyst (“dummy” variable) that codes each class numerically. In this study, samples belonging to the Normal category were described by the dependent vector (1 0) and samples belonging to the DFD category by the vector (0 1). A value of 1 was assigned when the sample belonged to the class and 0 when it did not. Usually, the Y matrix consists of integers. The results of the regression are Y values close to those that indicate each category. Under these assumptions, it is possible to use traditional regression methods to perform classification and compute a calibration model relating the matrix of predictors and this dummy matrix of responses. The core of the PLS-DA approach is the use of partial least squares regression, which performs bilinear decomposition of both the X and Y spaces, under the assumption that a relationship between the two internal spaces exists, to compute the model parameters. The optimum number of PLS factors (LVs) for the models was selected by leave-one-out cross-validation.

Discriminant models were evaluated according to the percentage of samples correctly classified during calibration development and subsequent cross-validation. Calibrations with the highest coefficient of determination of calibration (R^2^c) and cross-validation (R^2^cv) and the least PLS latent variables (LVs) were selected.

Moreover, the sensitivity (SE) and specificity (SP) of the discriminant models were calculated according to the following expressions, combining the number of true positives (TP), true negatives (TN), false positives (FP), and false negatives (FN) obtained in cross-validation:

SE = TP/(TP + FN)

SP = TN/(TN + FP)

SE indicates the proportion of samples belonging to one specific group that are correctly identified, while SP indicates the correct identification of samples that do not belong to that group. Results are expressed as R^2^cv and RMSECV statistics from cross-validation and the percentage of SE and SP in the Normal and DFD categories for calibration and cross-validation of the different mathematical treatments applied and the total sample set or AV, RG, and RE sample sets.

##### Soft Independent Modelling of Class Analogies (SIMCA)

In contrast with PLS-DA, for problems such as those concerning the fast classification of quality meat online involving real-time decision making, we considered it interesting to assess whether unknown samples would be compatible with the model of a specific category, which is exactly what SIMCA, a supervised pattern recognition method, does.

In SIMCA, each group is independently modelled by means of a PCA per category (Normal PCA and DFD PCA) and sample set (Total, AV, RG, and RE), thus determining the centroid and dispersion of samples in each category. The dimension of the individual model is given by the number of principal components (PCs) that are taken into account. One sample can be classified into only one group or into several groups, or as not belonging to any group, depending on the distance of the sample from the center of the model (leverage), reported according to the placement of the sample projected on the PCs and the distance of the sample from the model defined by the PCs (S distance).

For model evaluation, we examined the percentage of samples that were correctly classified. If a sample belonged to two or more groups, it was classified in the one with the lowest leverage and S distance values: (i) the sample was correctly classified according to the predefined categories, or (ii) the sample did not fit into any of the categories. The SIMCA results are expressed as the percentages of sensitivity and specificity of the model.

#### 2.4.2. Quantitative NIR Analysis of Quality Traits: Partial Least Squares (PLS)

Partial least squares regression (PLSR) quantitative prediction models were developed for the analyzed quality traits. PLSR models were calibrated using the nonlinear iterative partial least squares (NIPALS) algorithm, and the performance models were evaluated using leave-one-out full internal cross-validation. Spectral data subjected to PLS produced a new and smaller set of variables called latent variables (LVs); the optimal number was calculated as the number of LVs after which the standard error of cross-validation (SECV) no longer decreased substantially. The goodness of predictive models was assessed, selecting the model that had the optimal combination of the highest R^2^c and R^2^cv values, the minimum RMSECV, and the least PLS LVs considered to be optimal; this result was validated using the explained variance test in the Unscrambler X vs. 10.5 (CAMO^®^, Trondheim, Norway) software package.

According to Saeys et al. [18], a value of R^2^ between 0.66 and 0.8 indicates an approximate quantitative prediction, whereas a value between 0.81 and 0.90 indicates a good prediction. Calibration models with R^2^c > 0.90 are considered excellent. Moreover, to assess the practical utility of the prediction models, the relative predictive deviation (RPD) and range error ratio (RER) were calculated and considered as additional criteria to determine the prediction capacity of each model. The RPD is calculated as the ratio of the standard deviation (SD) to the RMSECV of a given trait. Prieto et al. [10] considered that RPD between 2 and 2.5 means approximate quantitative predictions are possible and can be applied to meat products and most agricultural materials. RER values < 3 indicate low predictive capability, models with RER between 3 and 10 have low to moderate practical utility, and RER values > 10 indicate good practical utility [19].

## 3. Results and Discussion

### 3.1. Meat Quality Traits

Table 1 shows the descriptive statistics related to meat quality traits from the whole sample population included in the study. The results include a mean pH24 of 5.68 ± 0.30, which falls within the normal range for meat (5.4 to 5.8) [4]. However, the pH range found in this study was wider (5.31 to 6.91), probably due to the different characteristics of the breeds included and because 18 samples (seven from AV, seven from RG, and four from RE) showed pH24 ≥ 6.0. As mentioned above, these samples were considered DFD and they contributed to the increased variability of all quality traits analyzed, and therefore to the goodness of the developed quantitative models.

Color parameters also showed wider ranges than those previously reported in the literature [11,20,21,22]. Whereas the normal range for L* in beef has been considered to range from 30 to 45, our results showed a wider range, from 23.85 to 50.77; meanwhile, a* varied from 4.63 to 27.02 and b* from 3.27 to 21.14. In this sense, previous studies conducted by our group [7] with this sample set showed significant differences among breeds for pH24 (*p* ≤ 0.05), L* (*p* ≤ 0.001), and a* (*p* ≤ 0.001). Significant differences (*p* < 0.001) were also found when comparing Normal vs. DFD samples from each breed for pH24, color, and drip loss.

De Marchi et al. [23] suggested that coefficient of variation (CV) values ranging from 6 to 19% indicate the existence of exploitable variability in the development of calibration models. Our results showed covariance values over 11% for all quality traits analyzed, except for pH24. Low covariance values for pH were also reported in studies related to the use of NIR in beef [12,21,24].

### 3.2. NIRS Classification (Normal and DFD)

In the Total sample set, after adopting pH24 ≥ 6.0 [5] as an objective criterion to identify DFD meats, 111 samples were identified as Normal and 18 as DFD, with a different incidence rate for each breed. The results revealed that the AV breed had 43 Normal meat samples (pH24 < 6.0) and 7 DFD meat samples (pH24 ≥ 6.0), which means a 14% incidence of DFD meat. In the case of RG, 30 samples were identified as Normal and 7 as DFD (18.9%). In the RE breed, 38 samples were considered as normal and 4 as DFD meat (9.5%). These results are in agreement with previous studies [6] reporting an incidence of around 10% of beef production in Australia and the United Kingdom, although some studies including calves showed DFD percentages of up to 43%, considering pH24 ≥ 5.8 [25].

Thus, after defining the samples as Normal or DFD based on pH ≥ 6, NIR spectral classification models (PLS-DA and SIMCA) were developed to detect DFD samples for decision making in real time.

#### 3.2.1. Partial Least Squares-Discriminant Analysis (PLS-DA)

Discriminant models for DFD and Normal meat for Total, AV, RG, and RE sample sets were developed by means of PLS-DA with the full (1000–2500 nm) or bounded (1000–1800 nm) spectral range and after applying different pretreatments. The results are shown in Supplementary Tables S1–S4, respectively, and the best-fitting prediction equations (selected according to calibration and cross-validation statistics) are summarized in Table 2. The table gives the calibration and cross-validation statistics and the sensitivity and specificity for classification of Normal and DFD meat (Longissimus thoracis).

The best discriminant models were obtained with the absorbance (log 1/R) spectra without any other pretreatment and using the full spectral range (1000–2500 nm) for the Total sample set and the bounded spectral range (1000–1800 nm) for AV, RG, and RE sample sets. Prieto et al. [24] also reported better absorbance results in a study on NIR prediction of pH24 values in Longissimus thoracis from young cattle.

The discrimination between Normal and DFD was successful for AV and RG sample sets, showing SE values higher than 90% (97.67 and 93.33%, respectively) and SP values of 100% and 71.43%, respectively, which means that the models were accurate and could be used as an alternative method to classify DFD beef. However, inaccurate SP results were found in the Total sample set (5.56%). RE showed intermediate values (SE = 95% and SP = 50%).

Our results are in accordance with those of Prieto et al. [13], who correctly classified 95 and 88% Normal and 95 and 93% dark cut in non-bloomed and bloomed beef samples, respectively, using PLS-DA based on Vis-NIR spectra. Similarly, Reis and Rosenvold [12], in a study using classification with PLS-DA predicted values between high and normal pH24, found that 93% of all animals sampled (bulls, heifers, cows, and steers) with pH24 ≥ 5.8 were correctly classified; when bulls were excluded, the rate of correctly classified samples was 90% for high pH24 and 89% for normal pH24 in the validation dataset. Nubiato et al. [14] proved that other non-destructive techniques, such as hyperspectral imaging, could be applied to Normal and DFD classification of Nellore beef samples using the linear discriminant analysis (LDA) model. These authors reported that 73 out of 78 samples were correctly classified, resulting in an overall accuracy of 93.6%, a sensibility of 94.0%, and a specificity of 90.9% using the full-range spectrum (928–2524 nm) for both pretreatment absorbance and reflectance combined with the second derivative.

Our values are slightly lower than those reported in the literature, probably due to the lack of information from the visible part of the spectrum (350–1000 nm). Although the potential use of NIRS technology seems clear, Lomiwes et al. [26] concluded that qualitative PLS models used for classification into two categories (Normal pH24 ≤ 5.7 or DFD pH24 > 5.7) were unsuccessful. In fact, a significant proportion of high pH24 meat was incorrectly classified as normal, suggesting that NIR was not able to differentiate between high and normal pH24 muscle using the combined spectra of different animal classes. These authors proposed that stronger models could be obtained by modelling for each animal class independently. This assumption agrees with the poorer results in this study for the Total sample set than the individual breeds.

In the RE breed, cross-validation SE and SP values for Normal and DFD meat were lower than those found for AV and RG, probably because RE is genetically more different from the other two breeds, also showing important differences in meat quality traits [27]. This could also be the reason for the worse results obtained using the Total sample set, since these genetic and quality differences were also observed at the spectral level. Remarkable differences in absorbance were found in mean spectra among the three breeds (Supplementary Figure S1) with AV and RG breeds showing more similar spectra compared to RE, probably due to their lower genetic difference.

As mentioned above, previous studies by our group [7] compared quality traits of DFD and Normal beef from AV, RG, and RE breeds and found that, in general, DFD meat from AV and RG showed differences in quality (reduced loss of juice, darker color, and faster growth of mesophilic microorganisms during aging) with respect to Normal meat, but not RE. This may be due to differences in postmortem muscle metabolism among breeds. In fact, in AV and RG, the DFD meat was darker, while for RE the meat was the most red. In addition, DFD meat showed a higher growth rate of mesophilic microorganisms than Normal meat in both AV and RG breeds (at 7 and 14 days of aging), while in RE meat the growth rate was lower, and no significant differences were found between Normal and DFD meats [7]. These quality differences among breeds were also noted by other authors [28], who associated genotype with the occurrence of DFD meat.

**Figure 2:**
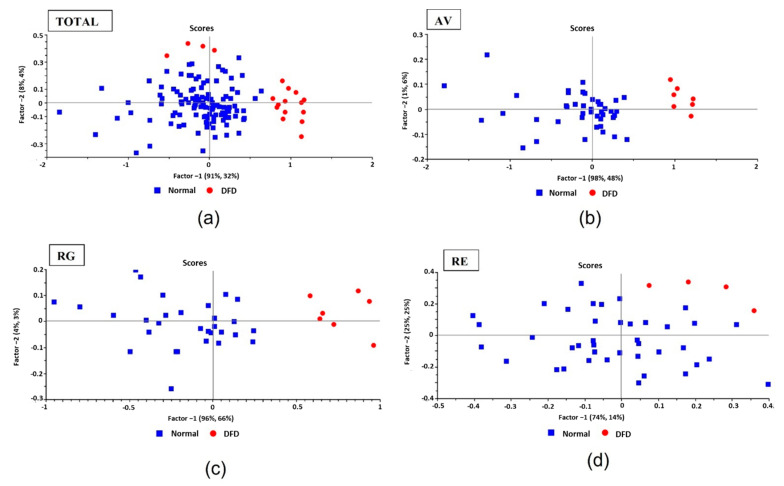
PLS-DA analysis after absorbance (log 1/R): 2D scatter plot of scores of total meat spectra data from: (**a**) Total (1000–2500 nm); (**b**) Asturiana de los Valles (AV; 1000–1800 nm); (**c**) Rubia Gallega (RG; 1000–1800 nm); and (**d**) Retinta (RE; 1000–1800 nm) sample sets. Graphical representation of PC1 (91%, 32%; 98%, 48%; 93%, 66%; and 74%, 14%, respectively) vs. PC2 (8%, 4%; 1%, 4%; 3%, 3%; and 25%, 25%, respectively). Red dots: DFD samples; blue squares: Normal samples.

In addition, the RE breed also showed a lower incidence (9.5%) of DFD meat, and these samples did not differ much in terms of quality traits from Normal meat (lightness, CRA, etc.). This phenomenon is also graphically visualized in Figure 2, which represents the absorbance (log 1/R) data matrix (2D) reduced to a coordinate axis system, where each sample is defined by the corresponding score for each PLS component. In AV and RG breeds (Figure 2b,c), when the whole sample set is represented on the XY plane according to the scores for PLS components 1 and 2, two different clusters can clearly be observed, with the samples located together being more similar in terms of their spectral characteristics. The samples belonging to the DFD group showed the highest values for PLS component 1. However, in RE (Figure 2d) the DFD samples are closer to the Normal ones and a similar distribution is also observed when analyzing the Total sample set (Figure 2a).

Several authors have studied the potential use of NIRS technology as a faster, non-destructive, and efficient tool to classify meat with different quality traits in other species. Indeed, NIRS technology was feasibly applied to intact chicken breast fillets to classify them as pale, soft, and exudative (PSE), DFD, or Normal meat [29]. Additionally, Monroy et al. [30] used NIRS to discriminate pork meat quality classes as red, soft, and exudative (RSE); pale, firm, and non-exudative (PFN); and reddish-pink, firm, and non-exudative (RFN), obtaining correct classifications with 79% accuracy.

**Figure 3:**
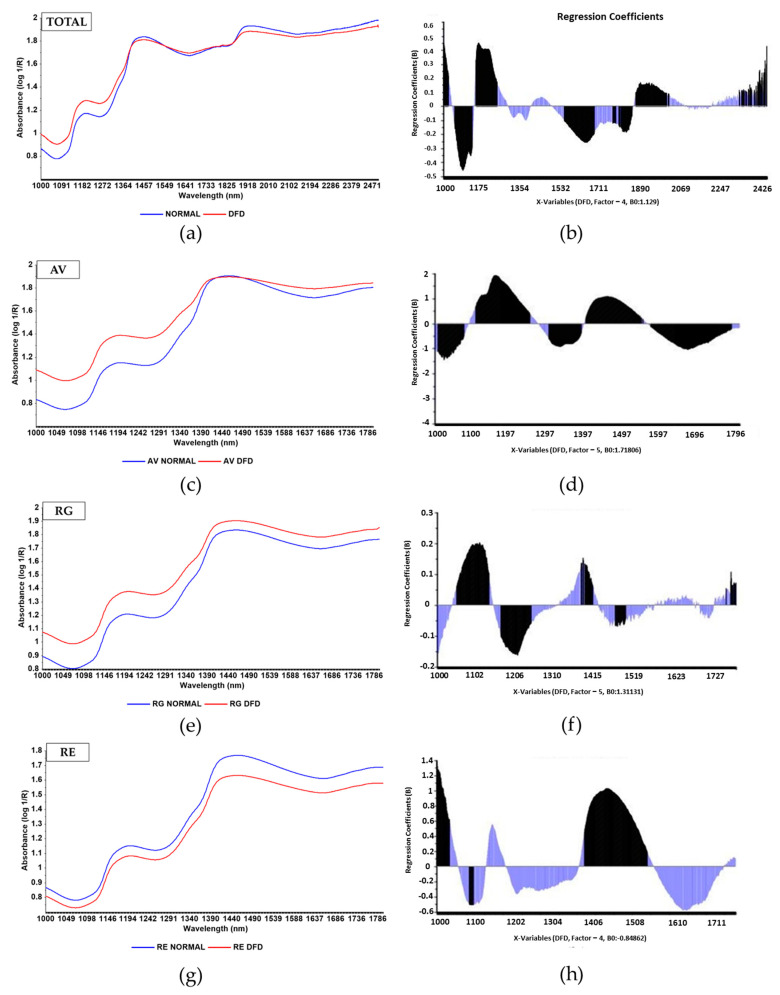
Left: Mean absorbance (log 1/R) spectra of Normal (blue) and DFD (red) meat samples from: (**a**) Total sample set after averaging 110 Normal and 18 DFD spectra (1000–2500 nm); (**c**) purebreed Asturiana de los Valles (AV) after averaging 43 Normal and 7 DFD spectra (1000–1800 nm); (**e**) purebreed Rubia Gallega (RG) after averaging 30 Normal and 7 DFD spectra (1000–1800 nm); and (**g**) purebreed Retinta (RE) after averaging 40 Normal and 4 DFD spectra (1000–1800 nm). Right: PLS-DA analysis after absorbance (log1/R): graphical representation of regression coefficients (B) of wavelengths in DFD meat spectra data from: (**b**) total (1000–2500 nm), (**d**) AV (1000–1800 nm); (**f**) RG (1000–1800 nm), and (**h**) Retinta (1000–1800 nm) sample sets. Significant variables are highlighted in black.

Regarding spectral features, Figure 3 shows the mean absorbance (log 1/R) spectra at 1000–2500 nm for the total sample set and at 1000–1800 nm for the AV, RG, and RE sample sets, and a graphical representation of regression coefficients of DFD from PLS-DA at 1000–2500 nm for the Total sample set and 1000–1800 nm for the AV, RG, and RE sample sets from Normal and DFD meat samples. In this figure, important differences are noted among breeds around 1100 and 1500 nm, which correspond to the wavelengths with the highest weight (regression coefficients) in PLS-DA (Figure 3b,d,f,g).

For AV and RG samples, the 1200 and 1330–1410 nm wavelengths were important variables in the PLS-DA classification model. On the contrary, RE samples did not show high regression coefficient values at those wavelengths; hence, this breed was different to the other two breeds in terms of DFD meat characteristics. The Total sample set showed intermediate values. Regarding the wavelengths selected in our study, in general, there was a predominance of wavelengths between 1100 and 1200 nm, corresponding to the second overtone of the C-H bonds, and 1330–1410 nm, corresponding to the first overtone combinations of C-H bonds [31]. The energy absorption at these wavelengths could be due to the hydrocarbon chains of fatty acids in the fat; hence, it would be expected that the CIE-L* color index estimation would be determined, to a large extent, by the amount of fat in the meat samples, as it is an important quality trait that is affected in DFD meat [7]. In fact, Page et al. [32] observed that muscle pH is inversely correlated with the fattiness of the carcass, which in turn may be directly correlated with intramuscular fat. Moreover, the band around 1500 nm (first overtone of N-H bonds) would be associated with the protein content [11], which is directly related to the heme pigment content of the meat and therefore to the CIE color index (also modified in DFD meat), which, as described above, was more associated with RE; hence, the regression coefficient values at that wavelength were higher for this breed.

#### 3.2.2. SIMCA Analyses

SIMCA analyses were performed using the spectral ranges and mathematical treatments, which produced better results in the above PLS-DA for each sample set. Absorbance (log1/R) of 1000–2500 nm for the Total sample set and 1000–1800 nm for the AV, RG, and RE sample sets were used (Table 3).

Calibration models for the classification of Normal samples (Total, AV, RG, and RE) resulted in rather good sensitivity values (94.6, 93.02, 96.6, and 97.5%, respectively), but the specificity decreased considerably in the cross-validation process. However, in the case of DFD, class models showed perfect sensitivity (100% in all datasets) and specificity was almost perfect for AV, RG, and RE (100, 100, and 90%, respectively) while it was very low for the Total sample set (19.8%). Therefore, as observed above in the PLS-DA results, using the Total sample set led to worse findings than the individual breeds, even though the model for the DFD categories in RE had significantly lower specificity than the other two. This could be explained by the inherent differences in samples coming from these breeds, as stated above, mainly due to genetic traits [7]. However, generally, the models for the DFD meat category showed sensitivity and specificity values of more than 90% for the cross-validation of samples from the three breeds.

Figures S2–S5 show the SIMCA plots of sample-to-model distance (Si) and sample leverage (Hi) for a given model. The plots include the class membership limits for both statistics: projection of samples to the Normal PCA model and to the DFD PCA model of meat spectral data from Total, AV, RG, and RE sample sets, respectively.

### 3.3. NIR Predictive Models for Quality Traits

Calibration models for the different quality traits related to DFD were developed for the Total sample set, and the best results are summarized in Table 4. The complete information on the best models developed within the full spectral range (1000–2500 nm) and the bounded spectral range (1000–1800 nm) for all variables under analysis at different postmortem times are shown in Supplementary Tables S5 and S6, respectively.

The best predictive models were obtained for color parameters a*, hue, and chroma after 60 min of blooming, showing coefficients of determination of calibration (R^2^c) and cross-validation (R^2^cv) over 0.87 and 0.82, respectively; RER > 8.9 and RPD > 2.2, and slightly lower for L* and b*, with R^2^c > 0.76, R^2^cv > 0.68, RER > 9, and RPD > 1.7. The rest of the prediction models for quality traits were less accurate.

It is important to note that pH_24_, drip loss, and color parameters L*, a*, and b* showed better results when the full spectral range was included; for the other quality traits (hue and chroma) the calibration results improved when the bounded spectral range (1000–1800 nm) was used. The number of LVs considered by the PLSR to build the calibration equations for the quality traits ranged from 4 to 8, in agreement with previous works [10,21]. This range can be considered adequate to avoid overfitting, which would lead to unlikely optimistic predictions.

With respect to the mathematical pretreatments, unlike what happened in the classification models where the absorbance spectra produced the best results, for quantitative predictions the optimal findings were obtained after SNV-DE with or without the Savitzky–Golay (SG) first-order derivative, which enhanced model accuracy in most cases.

Prieto et al. [11] stated: “Meat is a very heterogeneous material, subject to continuous modifications and largely influenced by environmental conditions and processing procedures, therefore meat quality traits are affected by many factors such as animal breed, type of muscle, sampling location, slaughter and ambient conditions, ageing and the type of reference analysis performed. All these factors make it difficult to compare results from different studies as there are many possible sources of variation affecting the NIRS predictions”. Moreover, calibrations can be affected by the number of samples introduced, differences in NIR instruments, statistical and mathematical pretreatments, calibration methods, and validation procedures [33].

The ability of NIRS to predict meat color was tested in previous studies using ground and/or intact beef samples with varying results, with the predictions on intact samples generally being more accurate [21,34].

Comparing our results for color parameters with previous studies, we found that our L* predictive models (R^2^c > 0.76, R^2^cv > 0.69, RER = 9.58, and RPD = 1.8) were slightly worse than those previously reported [34,35], in which R^2^c and R^2^cv values were over 0.8 for prediction on intact beef, but similar to values reported by others [20,36,37], with R^2^c < 0.7.

With respect to a* (redness), our results (R^2^c = 0.87, R^2^cv = 0.818, RER = 9.063, and RPD = 2.3) were better than most of those previously published for intact beef [21,35,37].

Concerning the b* index, our results (R^2^c = 0.767, R^2^cv = 0.685, and RPD = 1.7) were better than those found in most previous studies with R^2^ values under 0.6 and RER under 7 [20,34,36]. However, to our knowledge, the highest regression coefficient for b* prediction was reported by Prieto et al. [24], with R^2^ over 0.9 and RPD = 2.51, who also showed coefficients of correlation up to 0.8 between absorbance data and L* and b* values in the ranges 1230–1400 and 1600–1710 nm. They associated these wavelengths with the absorbance of C–H bonds in fatty acids and hence to intramuscular fat, which could be responsible for the prediction of L* and b* values by means of NIR spectra, insofar as they were correlated with intramuscular fat content. The combined use of VIS-NIR spectral regions in some previous studies could have enabled better prediction of a* and b* in association with myoglobin [38].

Chroma and hue angle showed very good prediction coefficients, with R^2^c over 0.86 and R^2^cv over 0.82, which were even better than the values for the CIELAB coordinates, and the values previously reported for intact beef, with values over 8.9 for RER and over 2.3 for RPD [20,21,35]. Both parameters are better correlated with perceptual color attributes: the greater the chroma value, the more color saturation. On the other hand, larger hue angle values indicate less red color. Possibly the positive results in terms of the accurate prediction by NIR obtained in the present study can explain the use of chroma and hue, indicating the capacity of this technology to discriminate DFD meat.

The remaining quality traits under analysis in this study showed less accurate predictive results. pH24 showed poor calibration results with low R^2^c of 0.581 and R^2^_CV_ of 0.377 as well as RPD of 1.26, thus supporting previous studies on intact beef [21,34]. Prieto et al. [11] indicated that the limited predictive ability of NIR spectroscopy models for pH could be caused by the marbling content of the meat [39]. Variations in marbling content affect the repeatability of the reference method and jeopardize the predictability NIR spectroscopy, especially when pH measurements are performed and NIR spectra are collected in different muscle locations.

Our PLS model results for drip loss prediction were poor compared to other works [33,37], with R^2^c = 0.415, R^2^cv = 0.349, RER = 4.9, and RPD = 1.24. The high heterogeneity of beef samples [10] together with the low repeatability of WHC measurement have been indicated as potential reasons for the limited ability of NIR for this parameter [40].

## 4. Conclusions

This study demonstrated that NIRS technology may be useful as a tool for discriminating between Normal and DFD beef samples from autochthonous breeds. PLS-DA models developed with the bounded spectral range (1000–1800 nm) of absorbance (log 1/R) showed a higher accuracy for the classification of Normal and DFD meat in the AV and RG breeds, while for the RE breed and Total sample set, a decreased classification accuracy was noted. SIMCA models showed 100% sensitivity for the DFD meat of every breed, and 93.02% for AV, 96.6% for RG, and 97.5% for Normal meat, even though the specificity was lower. NIRS classification (PLS-DA and SIMCA) was more successful when the sample sets of each breed (AV, RG, and RE) were modelled independently compared to the Total sample set.

NIRS quantitative prediction models (with the absorbance spectra collected at 24 h postmortem) showed good results for L*, a*, b*, chroma, and hue after 60 min of blooming. These results might be improved by developing new calibration equations on a larger dataset including more biological variability.

The findings derived from this study indicate that NIRS technology could be satisfactorily applied as an internal quality control tool in the meat production chain.

## Figures and Tables

**Table 1:** Descriptive statistics of meat quality traits of Longissimus thoracis et lumborum meat of total sample set.

Variable	PM Time	N	Max	Min	Median	Mean	SD	CV
pH24	24 h	129	6.91	5.31	5.55	5.68	0.30	5.31
Drip loss	24 h	129	3.39	0.64	1.58	1.62	0.57	35.02
CIE-L*	60 min	117	50.77	23.85	38.95	38.47	4.66	12.13
CIE-a*	60 min	117	27.02	4.63	12.80	14.77	5.79	39.19
CIE-b*	60 min	117	21.14	3.27	11.54	11.24	2.99	26.62
Hue	60 min	117	58.06	21.74	40.76	38.73	11.71	30.24
Chroma	60 min	117	32.43	6.19	18.43	19.33	5.91	30.58

PM, postmortem; N, number of samples; Max, maximum; Min, minimum; SD: standard deviation; CV, coefficient of variation. Drip loss expressed as g water/100 g muscle. CIE-L*: lightness; CIE-a*: redness; CIE-b*: yellowness.

**Table 2:** PLS-DA results of the classification of Normal and DFD beef (Longissimus thoracis muscle) using best-fitting prediction equations for total, Asturiana de los Valles (AV), Rubia Gallega (RG), and Retinta (RE) sample sets in calibration and cross-validation obtained after spectral pretreatments.

					Calibration	Cross-Validation		
Sample Set	N	Pretreatment	Range (nm)	LVs	R^2^c	R^2^cv	SE	SP
Total	129	Absorbance (log1/R)	1000–2500	4	0.471	0.414	85.84	5.56
AV	50	Absorbance (log1/R)	1000–1800	6	0.879	0.8101	97.67	100
RG	37	Absorbance (log1/R)	1000–1800	5	0.856	0.811	93.33	71.43
RE	42	Absorbance (log1/R)	1000–1800	4	0.648	0.525	95	50

Total, Total sample set; AV, Asturiana de los Valles; RG, Rubia Gallega; RE, Retinta; N, number of samples; LVs, latent variables; R^2^c, coefficient of determination of calibration; R^2^cv, coefficient of determination of cross-validation; SE, % sensibility; SP, % specificity; Abs, absorbance; R, reflectance.

**Table 3:** Results of SIMCA analysis of NIR spectra data after absorbance (log 1/R) in cross-validation for both categories of beef (Normal vs. DFD) in Total, AV, RG, and RE sample sets.

					Normal			DFD	
Sample Sets	*n*	Pretreatment	Range (nm)	PCs	SE	SP	PCs	SE	SP
*Total*	129	Abs (log1/R)	1000–2500	2	94.6	55.6	2	100	19.8
*AV*	50	Abs (log1/R)	1000–1800	2	93.02	71.42	2	100	100
*RG*	37	Abs (log1/R)	1000–1800	2	96.6	42.8	2	100	100
*RE*	42	Abs (log1/R)	1000–1800	2	97.5	50	2	100	90

AV, Asturiana de los Valles; RG, Rubia Gallega; RE, Retinta; PCs, principal components; Abs, absorbance; SE, % sensitivity; SP, % specificity.

**Table 4:** Best-fitting NIR prediction equations (calibration and cross-validation statistics) for main quality traits of Longissimus thoracis muscle from Total sample set, including Asturiana de los Valles, Rubia Gallega, and Retinta samples.

						Calibration		Cross-Validation			
Variable	PM Time	Math Treatment	Range (nm)	N	LVs	RMSEC	R^2^c	RMSECV	R^2^_cv_	RER	RPD
pH24	24 h	SG 1,4,4,1 SNV-DE	1000–2500	128	4	0.194	0.581	0.239	0.377	6.695	1.263
Drip loss	24 h	Abs (log 1/R)	1000–2500	111	4	0.352	0.415	0.374	0.349	4.972	1.238
CIE-L*	60 min	SNV-DE	1000–2500	103	6	2.185	0.765	2.51	0.695	9.058	1.805
CIE-a*	60 min	SNV-DE	1000–2500	102	8	1.998	0.878	2.508	0.818	8.929	2.296
CIE- b*	60 min	SG 1,4,4,1 SNV	1000–2500	101	4	1.23	0.767	1.437	0.685	9.063	1.775
Hue	60 min	SG 1,4,4,1 SNV	1000–1800	103	7	3.28	0.924	4.06	0.887	8.947	2.967
Chroma	60 min	SG 1,4,4,1 SNV	1000–1800	104	6	2.098	0.867	2.43	0.825	10.800	2.387

PM, post-mortem; N, number of samples; LVs, latent variables; RMSEC, root mean square error of calibration; R^2^c, coefficient of determination of calibration; RMSECV, root mean square error of cross-validation; R^2^cv, coefficient of determination of cross-validation; RER, range error ratio; RPD, relative predictive deviation. Drip loss expressed as g water/100 g muscle. CIE-L*: lightness; CIE-a*: redness; CIE-b*: yellowness. Abs (log 1/R), absorbance; SNV-DE, standard Normal variate-detrending; SG 1,4,4,1, Savitzky-Golay first-order derivative, 4 smoothing left side points, 4 smoothing right side points, 1 polynomial order.

## Data Availability

Data is contained within the article or supplementary material.

## Supplementary Materials

The following are available online at https://www.mdpi.com/article/10.3390/foods11203274/s1: Figure S1. Mean absorbance (log1/R) spectra of meat samples from Longissimus thoracis et lumborum muscle from Asturiana de los Valles (blue), Rubia Gallega (green), and Retinta (red) breeds. Figure S2. SIMCA for meat spectra data and Total sample set after absorbance (log 1/R) (1000–2500 nm). Figure S3. SIMCA for meat spectra data from purebreed Asturiana de los Valles after absorbance (log 1/R) (1000–1800 nm). Figure S4. SIMCA for meat spectra data from purebreed Rubia Gallega after absorbance (log 1/R) (1000–1800 nm). Figure S5. SIMCA for meat spectra data from purebreed Retinta after absorbance (log 1/R) (1000–1800 nm). Table S1. PLS-DA results for classification of Normal and DFD beef (Longissimus thoracis et lumborum muscle) of total sample set in calibration and cross-validation obtained after spectral pretreatments. Table S2. PLS-DA results for classification of Normal and DFD beef (Longissimus thoracis et lumborum muscle) of Asturiana de los Valles sample set in calibration and cross-validation obtained after spectral pretreatments. Table S3. PLS-DA results for classification of Normal and DFD beef (Longissimus thoracis et lumborum muscle) of Rubia Gallega sample set in calibration and cross-validation obtained after spectral pretreatments. Table S4. PLS-DA results for classification of Normal and DFD beef (Longissimus thoracis et lumborum muscle) of Retinta sample set in calibration and cross-validation obtained after spectral pretreatments. Table S5. Best-fitting prediction equations (calibration and cross-validation statistics) for main quality traits of Longissimus thoracis et lumborum from Asturiana de los Valles, Rubia Gallega, and Retinta samples with full spectrum (1000–2500 nm). Table S6. Best-fitting prediction equations (calibration and cross-validation statistics) for main quality traits of Longissimus thoracis et lumborum from Asturiana de los Valles, Rubia Gallega, and Retinta samples with bounded spectral range (1000–18000 nm).
