# Supplementary material for: Use of Near-Infrared Spectroscopy to Discriminate DFD Beef and Predict Meat Quality Traits in Autochthonous Breeds
Source: Foods. 2022 Oct 20;11(20):3274. doi: 10.3390/foods11203274 (PMC9601313; doi:10.3390/foods11203274)
Supplement: Supplementary file 1 [file foods-11-03274-s001.zip › foods-1877945-supplementary.pdf]

Table S1. PLS-DA analysis: comparison of the correct classification rates (%) between Normal and DFD beef (*Longissimus Thoracis et lumborum* muscle) from *Total* samples sets in calibration and cross-validation obtained after the different spectral pre-treatments.

TOTAL					
**Pre-treatment**	**n**	**Range (nm)**	**LVs**	**Cal**	**CV**
**Abs (log 1/R)**	129	**1000-2500**	**4**	**47.1**	**41.4**
	129	1000-1800	3	39.2	33.8
SNV-DE	129	1000-2500	4	36.2	20.1
	129	1000-1800	6	32.7	23.9
SG1,4,4,1	129	1000-2500	2	43.1	36.9
	129	1000-1800	2	37.7	35.4
SG 1,4,4,1 SNV	129	1000-2500	4	26.3	12.3
	129	1000-1800	5	34.4	22.3
SG 1,4,4,1 SNV-DE	129	1000-2500	4	56.9	15.9
	129	1000-1800	6	43.2	23.7
SG2,5,5,2	129	1000-2500	2	42.0	36.3
	129	1000-1800	2	37.6	35.4
SG2,5,5,2 SNV	129	1000-2500	2	24.0	N/A
	129	1000-1800	2	6.1	N/A
SG2,5,5,2 SNV-DE	129	1000-2500	2	23.9	N/A
	129	1000-1800	2	6.1	N/A

n, number of samples**; LVs,** latent variables; **Cal**, Calibration sets; **CV,** cross-validation sets; **SNV-DE**, Standard Normal Variate- Detrending; **SG1,4,4,1,** Savitzky-Golay first order derivative, 4 smoothing left side points, 4 smoothing right side points, 1 polynomial order; **SG2,5,5,2**, Savitzky-Golay second order derivative, 5 smoothing left side points, 5 smoothing right side.

Table S2. PLS-DA analysis: comparison of the correct classification rates (%) between Normal and DFD beef (*Longissimus Thoracis et lumborum* muscle) from *Asturiana de los Valles* purebred in calibration and cross-validation obtained after the different spectral pre-treatments.

Asturiana de los Valles					
**Pre-treatment**	**n**	**Range (nm)**	**LVs**	**Cal**	**CV**
**Abs (log 1/R)**	50	1000-2500	6	80.1	72.1
	50	**1000-1800**	**6**	**87.9**	**81.01**
SNV-DE	50	1000-2500	5	84.2	63.2
	50	1000-1800	7	80.2	62.01
SG1,4,4,1	50	1000-2500	5	80.2	58.5
	50	1000-1800	7	81.5	73.9
SG 1,4,4,1 SNV	50	1000-2500	6	90.3	55.8
	50	1000-1800	7	78.4	59.4
SG 1,4,4,1 SNV-DE	50	1000-2500	3	83.5	61.8
	50	1000-1800	6	85.1	70.1
SG2,5,5,2	50	1000-2500	2	53.5	45.5
	50	1000-1800	2	52.9	49.9
SG2,5,5,2 SNV	50	1000-2500	2	51.2	24.06
	50	1000-1800	5	74.8	39.9
SG2,5,5,2 SNV-DE	50	1000-2500	2	47.9	12.6
	50	1000-1800	7	94.9	63.6

n, number of samples**; LVs,** latent variables; **Cal**, Calibration sets; **CV,** cross-validation sets; **SNV-DE**, Standard Normal Variate- Detrending; **SG1,4,4,1,** Savitzky-Golay first order derivative, 4 smoothing left side points, 4 smoothing right side points, 1 polynomial order; **SG2,5,5,2**, Savitzky-Golay second order derivative, 5 smoothing left side points, 5 smoothing right side.

Table S3. PLS-DA analysis: comparison of the correct classification rates (%) between Normal and DFD beef (*Longissimus Thoracis et lumborum muscle*) from *Rubia Gallega* purebred in calibration and cross-validation obtained after the different spectral pre-treatments.

Rubia Gallega					
**Pre-treatment**	**n**	**Range (nm)**	**LVs**	**Cal**	**CV**
**Abs (log 1/R)**	37	1000-2500	2	74.6	72.7
	37	**1000-1800**	**5**	**85.6**	**81.1**
SNV-DE	37	1000-2500	7	88.6	46.3
	37	1000-1800	5	58.2	33.9
SG1,4,4,1	37	1000-2500	2	70.7	66.9
	37	1000-1800	2	70.5	71.8
SG 1,4,4,1 SNV	37	1000-2500	7	99.6	12.6
	37	1000-1800	3	67.4	37.3
SG 1,4,4,1 SNV-DE	37	1000-2500	7	99.6	22.1
	37	1000-1800	3	68.3	30.7
SG2,5,5,2	37	1000-2500	2	78.2	62.9
	37	1000-1800	2	69.5	58.9
SG2,5,5,2 SNV	37	1000-2500	2	91.9	37.7
	37	1000-1800	2	71.1	56.3
SG2,5,5,2 SNV-DE	37	1000-2500	2	91.9	34.5
	37	1000-1800	2	71.1	53.2

n, number of samples**; LVs,** latent variables; **Cal**, Calibration sets; **CV,** cross-validation sets; **SNV-DE**, Standard Normal Variate- Detrending; **SG1,4,4,1,** Savitzky-Golay first order derivative, 4 smoothing left side points, 4 smoothing right side points, 1 polynomial order; **SG2,5,5,2**, Savitzky-Golay second order derivative, 5 smoothing left side points, 5 smoothing right side.

Table S4. PLS-DA analysis: comparison of the correct classification rates (%) between Normal and DFD beef (*Longissimus Thoracis et lumborum* muscle) from *Retinta* purebred in calibration and cross-validation obtained after the different spectral pre-treatments.

Retinta					
**Pre-treatment**	**n**	**Range (nm)**	**LVs**	**Cal**	**CV**
**Abs (log 1/R)**	42	1000-2500	3	54.5	33.8
	42	**1000-1800**	**4**	**64.8**	**52.5**
SNV-DE	42	1000-2500	4	45.9	12.7
	42	1000-1800	7	56.2	20.4
SG1,4,4,1	42	1000-2500	1	0.92	N/A
	42	1000-1800	1	0.6	N/A
SG 1,4,4,1 SNV	42	1000-2500	2	32.5	N/A
	42	1000-1800	2	8.8	N/A
SG 1,4,4,1 SNV-DE	42	1000-2500	1	32.1	N/A
	42	1000-1800	1	9.3	N/A
SG2,5,5,2	42	1000-2500	1	25.7	N/A
	42	1000-1800	1	2.1	N/A
SG2,5,5,2 SNV	42	1000-2500	1	26.8	N/A
	42	1000-1800	1	46.1	N/A
SG2,5,5,2 SNV-DE	42	1000-2500	1	26.8	N/A
	42	1000-1800	1	46.2	N/A

n, number of samples**; LVs,** latent variables; **Cal**, Calibration sets; **CV,** cross-validation sets; **SNV-DE**, Standard Normal Variate- Detrending; **SG1,4,4,1,** Savitzky-Golay first order derivative, 4 smoothing left side points, 4 smoothing right side points, 1 polynomial order; **SG2,5,5,2**, Savitzky-Golay second order derivative, 5 smoothing left side points, 5 smoothing right side; **N/A**, non-applicable.

Table S5. Best fitting prediction equations (calibration and cross-validation statistics) for the main quality traits of *Longissimus Thoracis et lumborum* from *Asturiana de los Valles*, *Rubia Gallega* and *Retinta* samples with the full spectrum (**1000-2500 nm**). The best prediction equations have been highlighted in bold font.

					Calibration		Cross-Validation			
**VARIABLE**	**PM time**	**Math treatment**	**n**	**LVs**	**RMSEC**	**R^2**	**RMSECV**	**R^2_{cv}**	**RER**	**RPD**
**pH24**	**24h**	**SG 1,4,4,1 SNV-DE**	**128**	**4**	**0.194**	**0.581**	**0.239**	**0.377**	**6.695**	**1.263**
**Drip loss**	**24h**	**Abs (log1/R)**	**111**	**4**	**0.352**	**0.415**	**0.374**	**0.349**	**4.972**	**1.238**
**CIE-L***	**60 min**	**SNV-DE**	**103**	**6**	**2.185**	**0.765**	**2.51**	**0.695**	**9.058**	**1.805**
**CIE-a***	**60 min**	**SNV-DE**	**102**	**8**	**1.998**	**0.878**	**2.508**	**0.818**	**8.929**	**2.296**
**CIE-b***	**60 min**	**SG 1,4,4,1 SNV**	**101**	**4**	**1.23**	**0.767**	**1.437**	**0.685**	**9.063**	**1.775**
**Hue**	60 min	SNV-DE	99	5	5.443	0.788	6.106	0.738	5.944	1.945
**Chroma**	60 min	SG 1,4,4,1 SNV-DE	101	3	2.87	0.75	3.299	0.677	7.955	1.752

PM, *post-mortem*; **n**: number of samples; **LVs:** latent variables; **RMSEC**: root mean square error of calibration; **R^2**:coefficient of determination of calibration; **RMSECV**, root mean square error of cross-validation **R^2_{cv}** :coefficient of determination of cross-validation; **RER**: range error ratio; **RPD**, ratio performance deviation; **Drip-loss** expressed as g of water/100g of muscle; **Abs (log 1/R)**: Absorbance; **SNV-DE**: Standard Normal Variate-Detrending; **SG 1,4,4,1:** Savitzky-Golay first order derivative, 4 smoothing left and right side points, 1 polynomial order; **SG 2,5,5,2**: Savitzky-Golay second order derivative, 5 smoothing left and right side points, 2 polynomial order.

Table S6. Best fitting prediction equations (calibration and cross-validation statistics) for the main quality traits of *Longissimus Thoracis et lumborum* from *Asturiana de los Valles*, *Rubia Gallega* and *Retinta* samples with the bounded spectral range (**1000-1800 nm**). The best prediction equations have been highlighted in bold font.

					Calibration		Cross-validation			
**VARIABLE**	**PM time**	**Math treatment**	**n**	**LVs**	**RMSEC**	**R^2**	**RMSECV**	**R^2_{cv}**	**RER**	**RPD**
**pH24**	24h	SG 1,4,4,1 SNV-DE	128	4	0.229	0.42	0.242	0.362	6.612	1.248
**Drip loss**	24h	Abs (log 1/R)	106	3	0.387	0.353	0.403	0.314	5.086	1.202
**CIE-L***	60 min	SG 1,4,4,1 SNV	107	3	2.707	0.6	2.846	0.566	7.184	1.512
**CIE-a***	60 min	SG 1,4,4,1 SNV-DE	107	7	1.665	0.913	1.984	0.879	10.452	2.868
**CIE-b***	60 min	SG 1,4,4,1	103	4	1.826	0.528	1.987	0.452	7.516	1.345
**Hue**	**60 min**	**SG 1,4,4,1 SNV**	**103**	**7**	**3.28**	**0.924**	**4.06**	**0.887**	**8.947**	**2.967**
**Chroma**	**60 min**	**SG 1,4,4,1 SNV**	**104**	**6**	**2.098**	**0.867**	**2.43**	**0.825**	**10.800**	**2.387**

PM, *post-mortem*; **n**: number of samples; **LVs:** latent variables; **RMSEC**: root mean square error of calibration; **R^2**:coefficient of determination of calibration; **RMSECV**, root mean square error of cross-validation **R^2_{cv}**:coefficient of determination of cross-validation; **RER**: range error ratio; **RPD**, ratio performance deviation; **Drip-loss** expressed as g water/100g of muscle; **Abs (log 1/R)**:Absorbance; **SNV-DE**: Standard Normal Variate- Detrending; **SG 1,4,4,1:** Savitzky-Golay first order derivative, 4 smoothing left and right side points, 1 polynomial order; **SG 2,5,5,2**: Savitzky-Golay second order derivative, 5 smoothing left and right side points, 2 polynomial order.

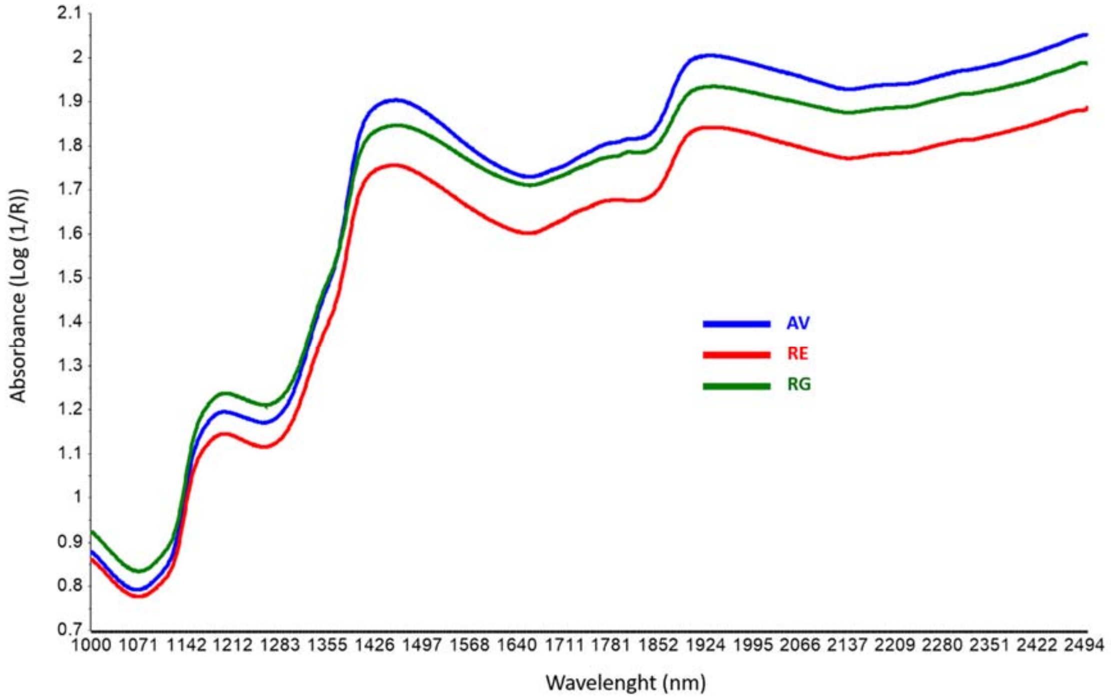

Figure S1. Absorbance (log1/R) mean spectra (1000-2500 nm) of meat samples from *Longissimus Thoracis et lumborum* muscle from *Asturiana de los Valles* (blue), *Rubia Gallega* (green) and *Retinta* (red) breeds.

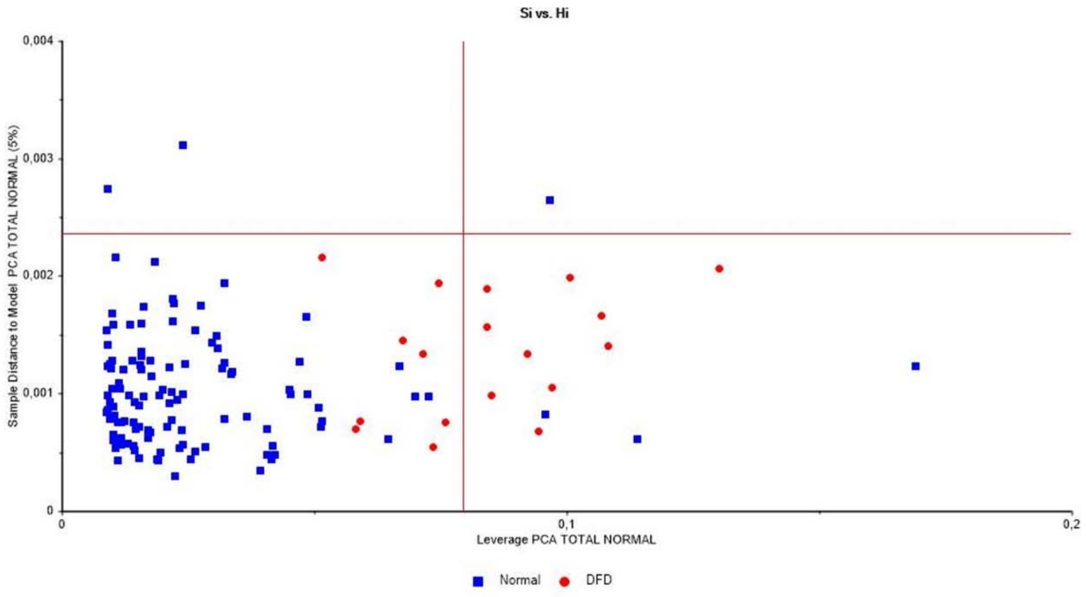

(a)

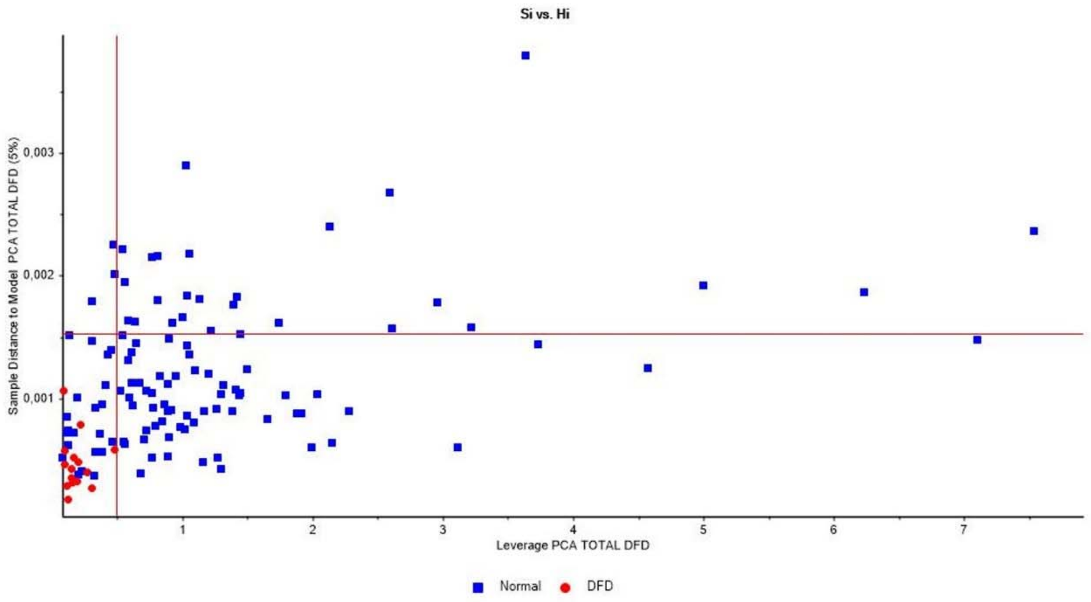

(b)

Figure S2. SIMCA for meat spectra data or *Total samples set* after Absorbance (log 1/R) (1000-2500 nm). This plot gives a view of both the sample-to-model distance (Si) and the sample leverage (Hi) for a given model. It includes the class membership limits for both statistics: **(a)** projection of samples to Normal PCA model and **(b)** projection of samples to DFD PCA model. Normal samples are in blue and DFD samples are in red.

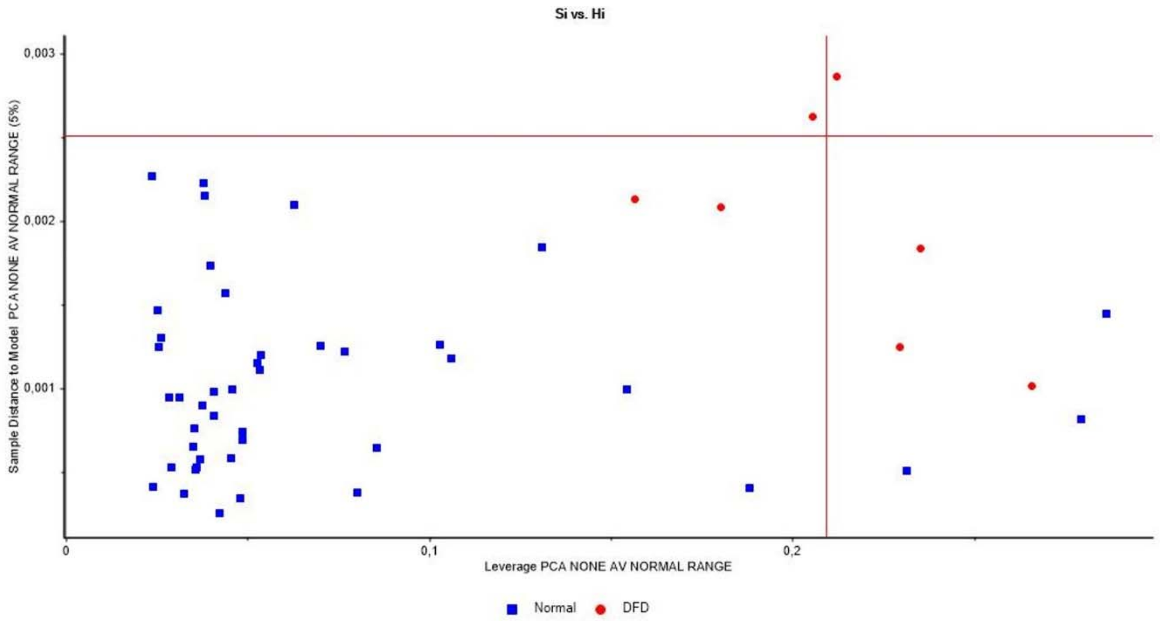

(a)

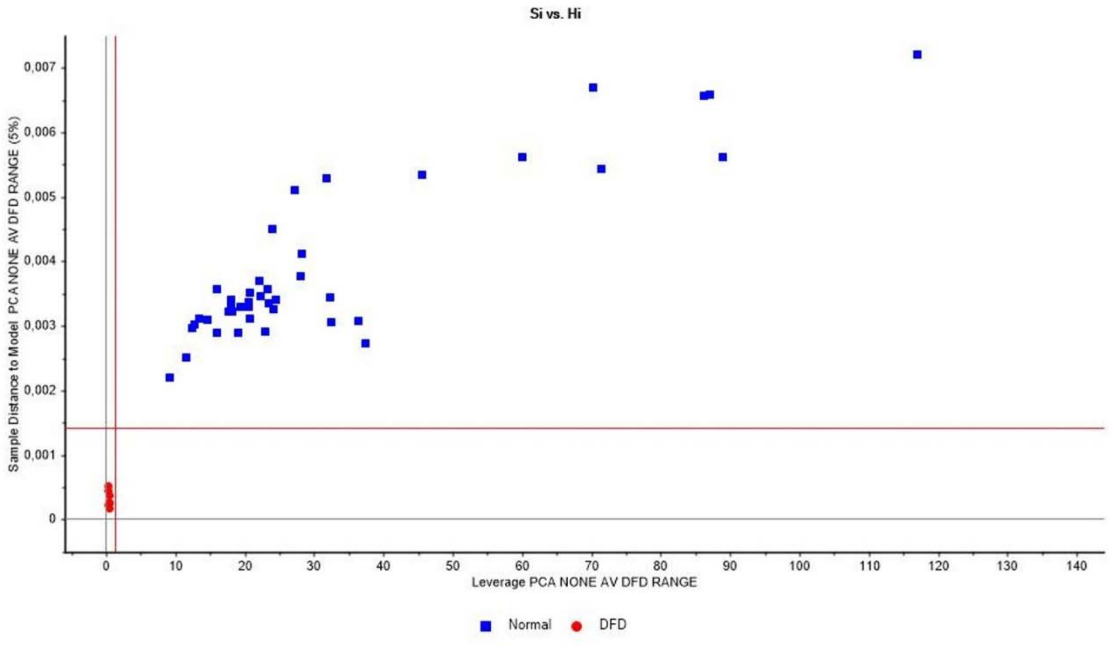

(b)

Figure S3. SIMCA for meat spectra data from *Asturiana de los Valles* purebred after Absorbance (log 1/R) (1000-1800 nm). This plot gives a view of both the sample-to-model distance (Si) and the sample leverage (Hi) for a given model. It includes the class membership limits for both statistics **(a)** projection of samples to Normal PCA model and **(b)** projection of samples to DFD PCA model. Normal samples are in blue and DFD samples are in red.

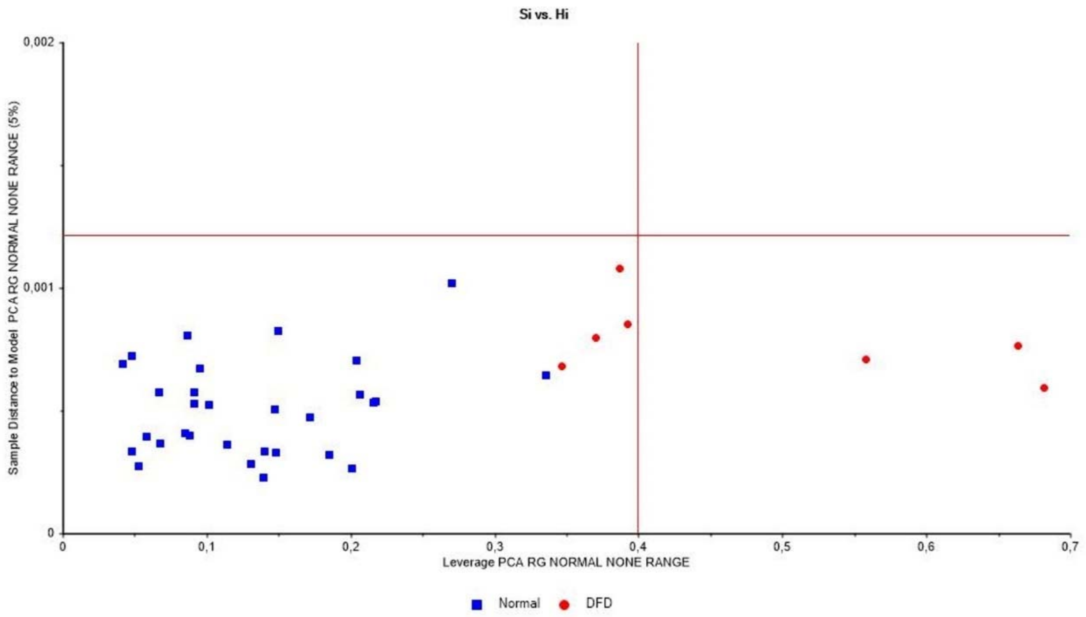

(a)

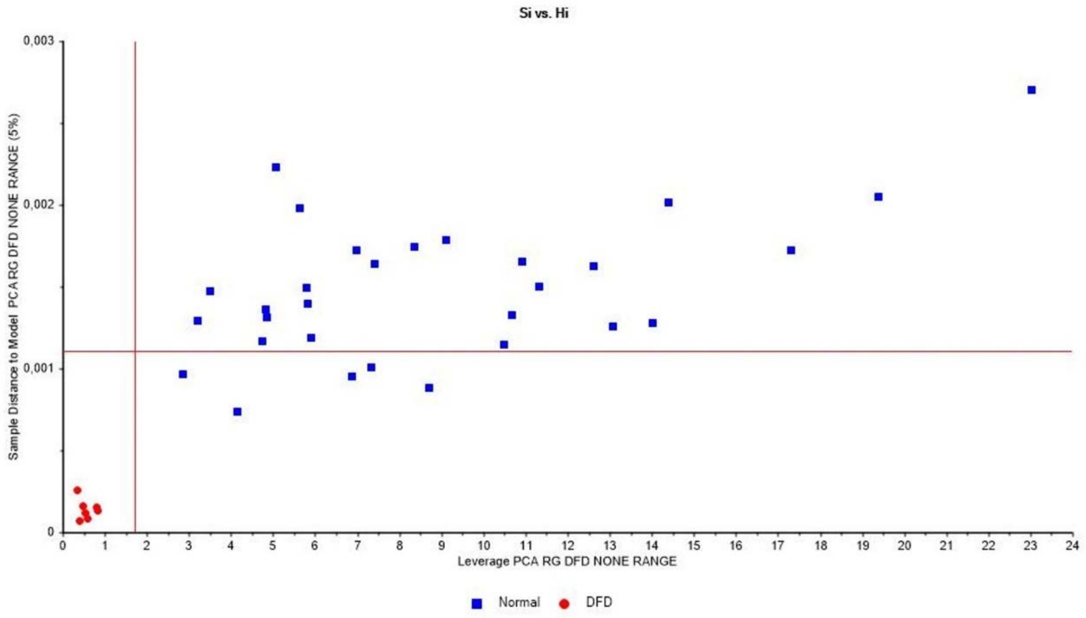

(b)

Figure S4. SIMCA for meat spectra data from *Rubia Gallega* purebred after Absorbance (log 1/R) (1000-1800 nm). This plot gives a view of both the sample-to-model distance (Si) and the sample leverage (Hi) for a given model. It includes the class membership limits for both statistics: **(a)** projection of samples to Normal PCA model and **(b)** projection of samples to DFD PCA model. Normal samples are in blue and DFD samples are in red.

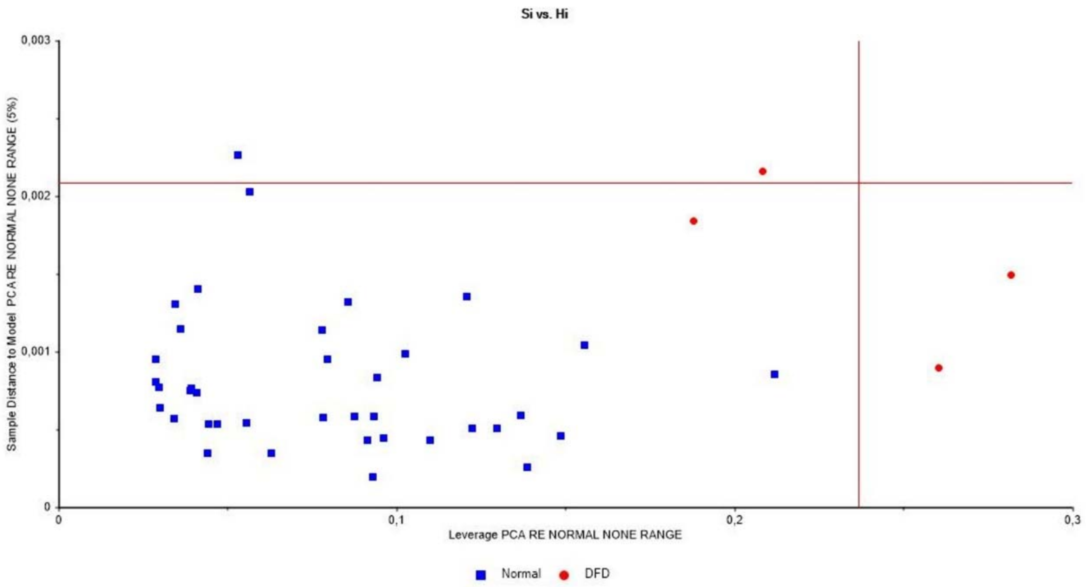

(a)

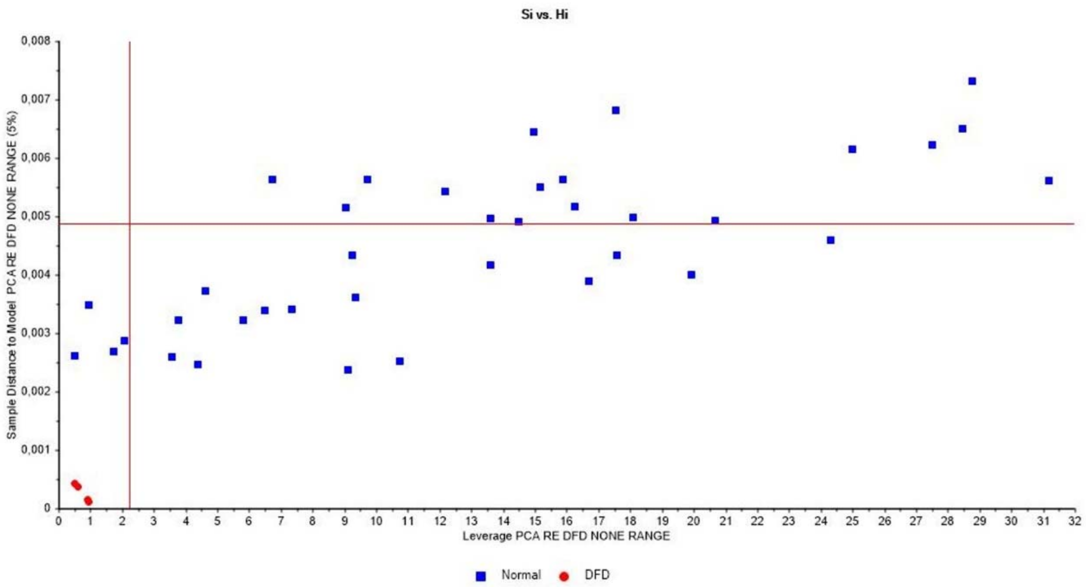

(b)

Figure S5. SIMCA for meat spectra data from *Retinta* purebred after Absorbance (log 1/R) (1000-1800 nm). This plot gives a view of both the sample-to-model distance (Si) and the sample leverage (Hi) for a given model. It includes the class membership limits for both statistics: **(a)** projection of samples to Normal PCA model and **(b)** projection of samples to DFD PCA model. Normal samples are in blue and DFD samples are in red.
